# The influence of infiltration feedback on the characteristic of banded vegetation pattern on hillsides of semiarid area

**DOI:** 10.1371/journal.pone.0205715

**Published:** 2019-01-15

**Authors:** Xiaoli Wang, Guohong Zhang

**Affiliations:** School of Mathematics and Statistics, Southwest University, Chongqing, 400700, P.R.China; Lanzhou University of Technology, CHINA

## Abstract

The formation and characteristics are two important topics in the study of vegetation pattern. In this paper, we focus on the influence of infiltration feedback of surface runoff on the formation and characteristics of banded vegetation pattern on hillsides of semiarid area. Firstly, it is shown that the infiltration feedback of surface runoff is an important formation mechanics of the banded vegetation pattern in semiarid area. Then it is found that the patterns formed within the reasonable parameter space are periodic both in space and time and the wave speed of the periodic vegetation stripes become larger with the increase of the infiltration feedback strength, while the wavelength is negatively correlated with the infiltration feedback strength. At last, it is shown that the mean productivity of grass pattern is lower than that of homogeneous steady state when the infiltration feedback is weak and it is larger than that with stronger infiltration feedback.

## Introduction

Vegetation patterns have been extensively studied by ecologists for a long time and different types of spatial patterns, such as bands, labyrinth, spots, stripes, and gaps, as well as plant rings have been observed in the arid and semiarid fields (see [1–6], and references therein). It has been identified that the formation and characteristic of vegetation pattern are affected by different factors, which include preferential infiltration in the vegetated area and run-off/run-on from bare zones toward vegetated ones [1, 3, 7], decreasing the evaporation and drainage in the vegetated area by the shading effect of biomass [2, 6], suction of water by the roots and transport of water in the soil [6, 8, 9]. Other factors, such as livestock overgrazing [10], climate variables [11–13], soil properties [14], precipitation interception [15, 16], toxic compounds [17–19], claimate change [20, 21] and the suction of roots [22] have also been identified as drivers of the different vegetation pattern formation.

Based on the view that vegetation pattern formation phenomenon involves symmetry breaking [1, 23], some independent mathematical models have been formed to investigate the vegetation patterns [1, 3, 6, 24–26]. In particular, Klausmeier [1] provides the following model to describe the relationship between plant communities and water-limited systems:

$$\left\{ \begin{array}{c} \frac{\partial w}{\partial t}=p-w-wn^{2}-v\frac{\partial w}{\partial x},\\ \frac{\partial n}{\partial t}=wn^{2}-\delta n+\nabla^{2}n, \end{array}\right.$$ (1)

where *w* is the water density and *n* is the biomass density; parameters *p*, *δ* are positive constants that denote the precipitation and the biomass loss rate, respectively; the term ∇^2^ *n* represents the diffusion of biomass, where ∇^2^ denotes the one or two dimensional Laplacian operator. The term *wn*^2^ accounts for the water uptake by plant. The term $v\frac{\partial w}{\partial x}$ describes the surface runoff which is proportional to the slope of terrain and *v* represents a constant downhill runoff flow velocity. Klausmeier predicts the key characteristics of stripe patterns, including the wavelengths and migration speed, which are in order-of-magnitude agreement with field observations.

Following Klausmeier′s model (1), Liu et al. [8] provide an extended model to study the positive feedback effects between the water and biomass on the vegetation spatial pattern formation:

$$\left\{ \begin{array}{c} \frac{\partial w}{\partial t}=p-w-wn^{2}-v\frac{\partial w}{\partial x}+\gamma \nabla^{2}\left(w-\beta n\right),\\ \frac{\partial n}{\partial t}=wn^{2}-n+\nabla^{2}n, \end{array}\right.$$ (2)

where the term *γ*∇^2^(*w* − *βn*) describes the suction of water by the roots and processes of water resource redistribution, which was first considered in vegetation-water model by Hardenberg et al. [6]. By numerical simulations, the changes of the wavelength, wave speed, as well as the conditions of the spatial pattern formation are investigated. Other related studies of Klausmeier′s model can also be referred to [1, 24, 27–33]. We point out that some studies on pattern formation were performed under the background of complex networks [34, 35].

**Fig 1:**
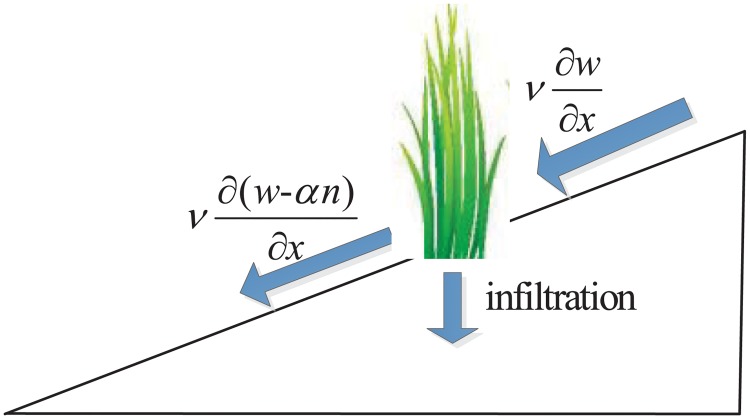
A model for the surface runoff on a slope. The term $v\frac{\partial w}{\partial x}$ model the surface runoff without biomass and $v\frac{\partial (w-\alpha n)}{\partial x}$ describes the drop of runoff in vegetated areas due to increased infiltration. Arrows indicate the direction of motion for surface water.

Inspired by the studies above, we consider an extended model of the Klausmeier′s model (1) with the following non-dimensional form:

$$\left\{ \begin{array}{c} \frac{\partial w}{\partial t}=p-w-wn^{2}-v\frac{\partial (w-\alpha n)}{\partial x},\\ \frac{\partial n}{\partial t}=wn^{2}-\delta n+\nabla^{2}n, \end{array}\right.$$ (3)

where the term $v\frac{\partial (w-\alpha n)}{\partial x}$ describes the increased infiltration due to the existence of vegetation, which was first considered in vegetation-water model by Hardenberg et al. [6]. From the viewpoint of biology, the surface runoff can be describes by $v\frac{\partial w}{\partial x}$ when there is no vegetation. While in vegetated areas, there exists a drop of runoff due to increased infiltration and then the surface runoff can be described by $v\frac{\partial (w-\alpha n)}{\partial x}$ (see Fig 1). The parameters *α* can also be seen as the cross-advection coefficient, which describes the positive feedback effect of biomass due to increased infiltration for surface runoff and the larger *α* refers to the stronger positive feedback effect. Then we always call the parameter *α* the infiltration feedback coefficient of surface runoff in the following sections.

So far, the research on an extended Klausmeier′s model with positive feedback effects of biomass due to increased infiltration of surface runoff is relatively few. Regular pattern formation is an intriguing natural phenomenon found in a broad range of ecosystems, such as arid ecosystems, wetland ecosystems, savannahs and mussel beds (see [4] for a review). It was reported that the spatial patterns may be an indicators for catastrophic shifts in response to changing conditions, such as climate change [26]. Then it is important to give a systematic mathematical and numerical analysis of the pattern characteristics for those ecosystems with regular patterns. In this paper, we would like to present a mathematical and numerical analysis for the development of spatial patterns, and investigate the positive feedback coefficient *α* how to affect the characteristics of the developing spatial patterns, which include the amplitude, wavelength and wave speed of the pattern solution.

The rest of this paper is organized as follows. In Section 2, we shall first study the effects of the positive feedback coefficient *α* on pattern formation by a linear stability analysis, and then obtain some results about critical advection strength, instability region. In Section 3, some results from numerical simulations for the system (3) are given with different (ecologically feasible) regions of the parameter space. Finally in Section 4, we give some conclusions and discussions.

## Materials and methods

This paper investigate the influence of infiltration feedback on the characteristic of banded vegetation pattern on hillsides of semiarid area. We first present a detailed linear stability analysis and then validated our theoretical findings through detailed parameter space identification and numerically solving the model.

## Results

### Homogeneous system

In the absence of diffusion, system (3) corresponds to the following spatial homogeneous system:

$$\left\{ \begin{array}{c} \frac{dw}{dt}=p-w-wn^{2},\\ \frac{dn}{dt}=wn^{2}-\delta n,\\ w\left(0\right)=w_{0}\geq 0,\;\;n\left(0\right)=n_{0}\geq 0. \end{array}\right.$$ (4)

Let *V* = *w* + *n* and take *c* = min{1, *δ*}. It follows from system (4) that

$$\frac{dV}{dt}\leq p-cV.$$

Then system (4) can be confined to the following region

$$D=\{\left(w,n\right):w\geq 0,n\geq 0,w+n\leq \frac{p}{c}\}.$$

Apparently *E*_0_ = (*p*, 0) is a trivial steady state of system (4), which corresponds to bare-soil state (no vegetation or desert state). The Jacobian matrix of system (4) at *E*_0_ takes the form of

$$J_{0}=(\begin{array}{cc} -1 & 0\\ 0 & -\delta \end{array}).$$

It is easy to see that the two eigenvalues of Jacobian matrix *E*_0_ are λ_01_ = −1 and λ_02_ = −*δ*. Hence the bare-soil state *E*_0_ is locally asymptotically stable.

Other possible positive equilibria include *E*_±_ = (*w*_±_, *n*_±_), where

$$\begin{array}{c} n_{+}=\frac{p+\sqrt{p^{2}-4\delta^{2}}}{2\delta },\;\;w_{+}=\frac{2}{p+\sqrt{p^{2}-4\delta^{2}}},\\ n_{-}=\frac{p-\sqrt{p^{2}-4\delta^{2}}}{2\delta },\;\;w_{-}=\frac{2}{p-\sqrt{p^{2}-4\delta^{2}}}. \end{array}$$

Then we have the following results about the positive equilibria which correspond to vegetation states.

**Proposition 0.1** *Let E*_±_ = (*w*_±_, *n*_±_).

(*i*) *If p* < 2*δ*, *system* (4) *has no positive equilibrium*;
(*ii*) *if p* = 2*δ*, *system* (4) *has a unique positive equilibrium E*_+_;
(*iii*) *if p* > 2*δ*, *system* (4) *has two positive equilibria E*_+_ *and E*_−_ *with n*_−_ < 1 < *n*_+_.

We now consider the stability of the positive equilibria *E*_±_ = (*w*_±_, *n*_±_). The Jacobian matrix at an equilibrium *E*_±_ is

$$J_{E_{\pm }}=(\begin{array}{cc} A_{\pm } & B_{\pm }\\ C_{\pm } & D_{\pm } \end{array}),$$

where

$$A_{\pm }=-n_{\pm }^{2}-1,\;\;\;B_{\pm }=-2\delta ,\;\;\;C_{\pm }=n_{\pm }^{2},\;\;\;D_{\pm }=\delta .$$ (5)

By direct calculations, we have

$$\text{det}\left(J_{E_{\pm }}\right)=A_{\pm }D_{\pm }-B_{\pm }C_{\pm }=\delta \left(n_{\pm }^{2}-1\right).$$

From the expressions of *n*_±_, we know that *n*_−_ < 1 < *n*_+_. Then the determinant $\text{det}(J_{E_{-}})<0$, which implies that the equilibrium *E*_−_ is a saddle and always unstable. The determinant $\text{det}(J_{E_{+}})>0$ and then the stability of the equilibrium *E*_+_ is determined by the sign of $\text{tr}(J_{E_{+}})$, where

$$\text{tr}\left(J_{E_{+}}\right)≔A_{+}+D_{+}=\delta -1-n_{+}^{2}.$$ (6)

Set

$$\bar{p}=\text{min}\{2\delta \sqrt{\delta -1},\;\;\frac{\delta^{2}}{\sqrt{\delta -1}}\}.$$

We can obtain the following theorem on the stability of *E*_+_.

**Proposition 0.2** *Assume p* > 2*δ*. *Then E*_+_ *is locally asymptotically stable if one of the following conditions is satisfied*

(*i*) *δ* < 2;
(*ii*) *δ* > 2 *and* $p>\bar{p}$.

*Further*, *if δ* > 2 *and* $p<\bar{p}$, *E*_+_ *is unstable and* $p=\bar{p}$ *is a Hopf bifurcation point where a family of small amplitude limit cycles emanate from E*_+_.

**Remark 0.3** *According to* [1], *we take δ* = 0.45 *or δ* = 0.045 (*δ* < 2) *in the following sections*, *which implies that E*_+_ *is locally asymptotically stable*.

### Conditions for pattern formation

#### Dispersion relation

Now we consider the temporal stability of the uniform state *E*_+_ to non-uniform perturbations

$$\begin{array}{cc} \left(\begin{array}{c} n\\ w \end{array}\right) & =(\begin{array}{c} n_{+}\\ w_{+} \end{array})+\varepsilon (\begin{array}{c} n_{k}\\ w_{k} \end{array})e^{\lambda t+ikx}+c.c.+O\left(\varepsilon^{2}\right), \end{array}$$ (7)

where λ is the growth rate of perturbations at time *t*, *i* is the imaginary unit and *i*^2^ = −1, *k* is the wave number corresponding to one space, and *c*.*c*. stands for the complex conjugate. Substituting (7) into (3) and neglecting all nonlinear terms in *n* and *w*, one finds the characteristic equation for the growth rate λ is determined by the determinant of the following matrix:

$$J=(\begin{array}{cc} \lambda -A_{+}+ivk & -i\alpha vk-B_{+}\\ -C_{+} & \lambda -D_{+}+k^{2} \end{array}).$$

Then the characteristic equation for the growth rate λ is

$$\lambda^{2}+\left(k^{2}+ivk-A_{+}-D_{+}\right)\lambda -A_{+}k^{2}+ivk\left(k^{2}-\alpha C_{+}-D_{+}\right)+A_{+}D_{+}-B_{+}C_{+}=0.$$

Thus, the dispersion relation is

$$\lambda =\frac{1}{2}(A_{+}+D_{+}-ivk-k^{2}+j\sqrt{\Phi +i\Psi }),$$ (8)

where *j* = ±1, and

$$\begin{array}{cc} \Phi & ={\left(k^{2}+A_{+}-D_{+}\right)}^{2}-v^{2}k^{2}+4B_{+}C_{+},\\ \Psi & =-2vk(k^{2}+A_{+}-D_{+}-2\alpha C_{+}). \end{array}$$

By some calculations, we can obtain from (8) the real part of λ

$$\text{Re}\lambda =\frac{1}{2}\{A_{+}+D_{+}-k^{2}+j{\left[\frac{1}{2}\left(\sqrt{\Phi^{2}+\Psi^{2}}+\Phi \right)\right]}^{\frac{1}{2}}\},$$ (9)

and the imaginary part of λ

$$\text{Im}\lambda =\frac{1}{2}\{-vk+j\text{sign}\left(\Phi \right){\left[\frac{1}{2}\left(\sqrt{\Phi^{2}+\Psi^{2}}-\Phi \right)\right]}^{\frac{1}{2}}\}.$$ (10)

It can be demonstrated that there does not exist Turing instability range in system (3). In fact, from a general linear analysis (see [36]), we know that the necessary conditions for Turing instability are given by:

$$\left\{ \begin{array}{cc} & A_{+}+D_{+}<0,\\ & A_{+}D_{+}-B_{+}C_{+}>0,\\ & A_{+}d_{n}+D_{+}d_{w}>0,\\ & {\left(A_{+}d_{n}+D_{+}d_{w}\right)}^{2}>4d_{n}d_{w}\left(A_{+}D_{+}-B_{+}C_{+}\right), \end{array}\right.$$ (11)

where *d*_*w*_ and *d*_*n*_ represent the diffusive coefficient of water and biomass, respectively. Since *d*_*w*_ = 0, *d*_*n*_ = 1 and *A*_+_ < 0, the third condition in (11) can not be satisfied, which implies the nonexistence of Turing patterns in system (3).

#### Critical slope for pattern formation

Although there is no Turing instability in system (3), we might expect a possible instability mechanism [1, 27]. The condition for a spatial mode to be unstable and thus to induce a pattern is that Reλ > 0 for some *k*. From Eq (9) we can derive the neutral surfaces, i.e. those surfaces in *v* − *k* − *p* space on which Reλ = 0. After a simple calculation we have

$$v=v\left(k,p\right)≜\sqrt{\frac{{\left(A_{+}+D_{+}-k^{2}\right)}^{2}\left(A_{+}k^{2}-A_{+}D_{+}+B_{+}C_{+}\right)}{\left(D_{+}+\alpha C_{+}-k^{2}\right)\left(A_{+}-\alpha C_{+}\right)k^{2}}}.$$ (12)

**Fig 2:**
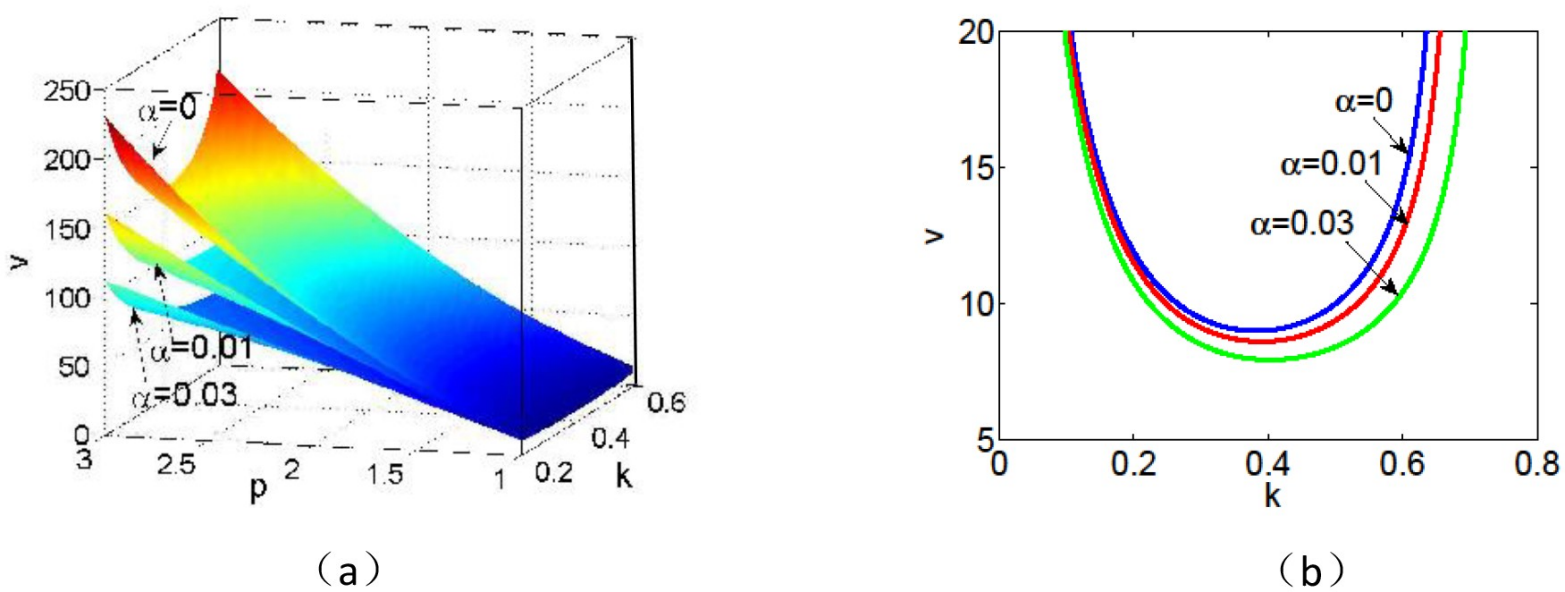
(a) Neutral surfaces *v* = *v*(*k*, *p*) defined in Eq (12) with the parameter values *v* = 182.5 and *δ* = 0.45; (b) Typical neutral curve *v* = *v*(*k*, 1) with the parameter values *v* = 182.5, *δ* = 0.45 and *p* = 1. One can see that the critical advection strength *v*_*c*_ decrease with the increase of infiltration feedback strength *α*.

It follows from (5) that *A*_+_ − *αC*_+_ < 0 and *A*_+_*k*^2^ − *A*_+_ *D*_+_+ *B*_+_ *C*_+_ < 0. Then a necessary condition for Eq (12) to hold is $0<k<\sqrt{D_{+}+\alpha C_{+}}$. A graph of neutral surfaces *v* = *v*(*k*, *p*) with different *α* is shown in Fig 2(a). For fixed *p* = *p*_0_, we can obtain the neutral curve *v* = *v*(*k*, *p*_0_) from (12). Fig 2(b) is a typical neutral curve, which is a cross section of Fig 2(a) with *p* = 1. It can be seen that the neutral curves *v* = *v*(*k*, 1) with different infiltration feedback strength *α* are convex in the range $0<k<\sqrt{D_{+}+\alpha C_{+}}$ and each of them has a unique minimum denoted by *v*_*c*_ at a non-zero value of *k*. Here the unique minimum *v*_*c*_ is defined as the critical advection strength, which gives the smallest advection strength for the formation of the stripe pattern. It is found that the critical advection strength *v*_*c*_ decreases with the increase of *α*, which indicates that the infiltration feedback of surface runoff helps to the formation of the stripe pattern on a hill with more gentle slope. For different precipitation *p*, we have the similar results. For fixed positive feedback level *α*, it can also be seen from Fig 2 that the critical advection strength *v*_*c*_ is positively correlated with the precipitation *p*, which indicates that the formation of stripe pattern in wetter area require steeper terrain(bigger *v*).

#### Instability region in *p* − *δ* plane

**Fig 3:**
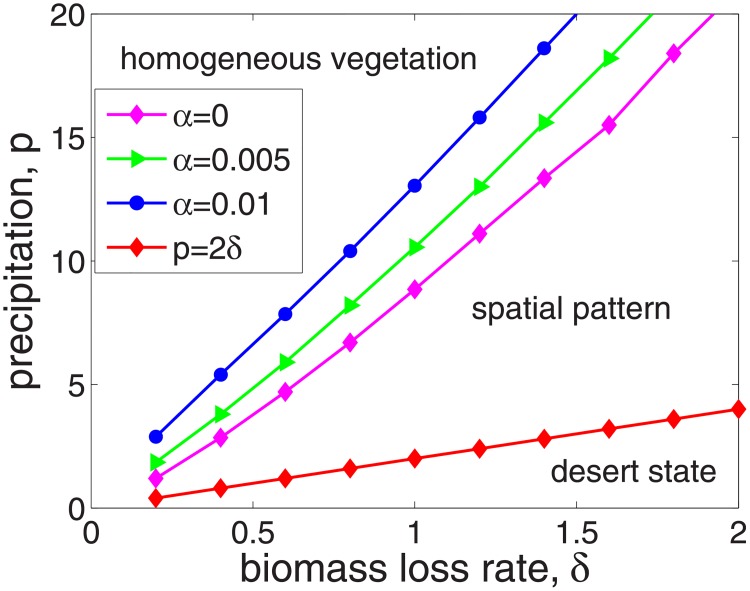
Effects of *α* on the instability region for equilibrium *E*_+_ on *p* − *δ* plane. The parameter values *v* = 182.5. One can see that stronger infiltration feedback (larger *α*) leads to larger instability region.

Now, we investigate the effects of the infiltration feedback of surface runoff *α* on the instability region. Here we fix parameter *v* = 182.5 and vary the values of *p* and *δ* and investigate the instability region in *p* − *δ* plane with different *α*. In detail, for each fixed *δ*, by computing the maximum of Reλ directly from formula (9), we can obtain Fig 3, which illustrates the results of a numerical analysis for stability within the *p* − *δ* space. It can be seen that the desert state occurs when the precipitation is too low; when the precipitation is too heavy, there is a homogeneous level of vegetation. The vegetation can survive with spatial pattern when the precipitation lies in the intermediate levels. One can see that the stronger positive feedback function (larger *α*) will lead to a larger parameter space region that a spatial pattern will emerge, which indicate that the infiltration feedback of surface runoff *α* has similar effect on the instability region with the suction effect by the roots as shown in paper [8].

#### Movement of stripe pattern

**Fig 4:**
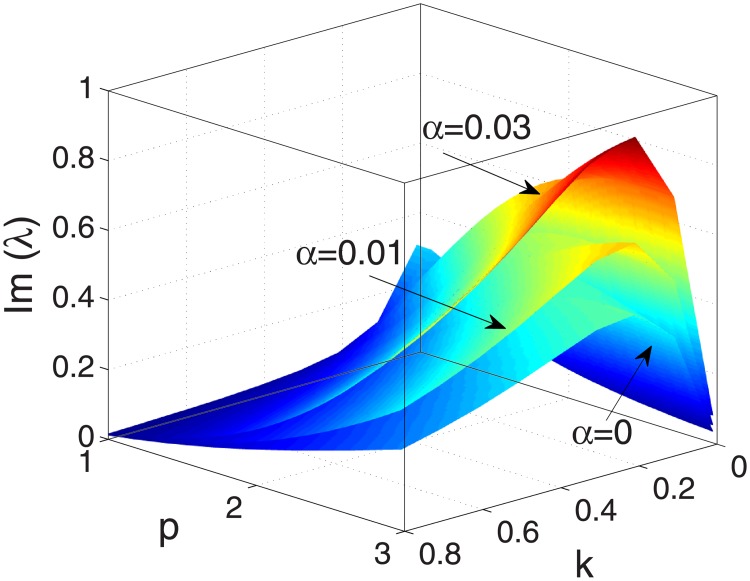
An illustration of imaginary values of eigenvalue λ = λ(*p*, *k*), given in Eq (10) for different *α*. The other parameter values are *v* = 182.5, *δ* = 0.45.

A key issue for biomass stripes is to determine the movement speed, and the way in which the speed vary with parameters. Here we fix parameter *v* = 182.5 and *δ* = 0.45 and vary the values of *α* to investigate the effect of *α* on the movement. At first we show that spatial pattern solutions are periodic in both space and time. Fig 4 shows imaginary values of eigenvalue λ = λ(*p*, *k*), obtained by Eq (10) for different *α*. We can find that Imλ > 0 for all $0<k<\sqrt{D_{+}+\alpha C_{+}}$ and 1 ≤ *p* ≤ 3, which means that the striped pattern predicted by the above calculations is not stationary, but rather moves over time [27]. Furthermore, one can note that Imλ increase with stronger infiltration feedback level *α*, which implies that the stripe patterns move faster with the increase of *α*.

### Existence of spatial pattern: Nonlinear analysis

We have investigated the existence and wave speed of the spatial patterns by linear analysis in the section above. In order to obtain more properties of the nonlinear system (3), it is necessary to do some nonlinear analysis.

Note that the spatial patterns on hillside emerge in the form of vegetation bands and migrate in uphill direction. So the patterns can be seen as periodic traveling wave solutions with the mathematical form *w*(*x*, *t*) = *U*(*z*), *n*(*x*, *t*) = *V*(*z*), where *z* = *x* + *ct* and *c* is the migration speed (see [24, 27–32]). Substituting these solution forms into (3) gives the ordinary differential equations (see [36])

$$\left\{ \begin{array}{c} c\frac{dU}{dz}=p-U-UV^{2}-v\frac{dU}{dz}+v\alpha \frac{dV}{dz},\\ c\frac{dV}{dz}=UV^{2}-\delta V+\frac{d^{2}V}{dz^{2}},\; \end{array}\right.$$ (13)

where *c* is now treated as a variable. The second-order ODEs system (13) predicts spatial periodic wave forms in the co-moving frame and can be written as a system of three first-order ODEs in the following standard way:

$$\left\{ \begin{array}{c} \frac{dU}{dz}=\frac{1}{v+c}\left(p-U-UV^{2}+v\alpha W\right),\\ \frac{dV}{dz}=W,\;\\ \frac{dW}{dz}=cW-UV^{2}+\delta V. \end{array}\right.$$ (14)

There is a steady-state solutions of the form (*U*, *V*, *W*) = (*w*_+_, *n*_+_, 0) with (*w*_+_, *n*_+_) being the spatially uniform steady-state solutions of system (3). The periodic traveling wave solutions will emerge in system (3) when Hopf instability occurs in system (14). The characteristic equation of linearized system (14) read as

$$\begin{array}{cc} \lambda^{3}+(\frac{n_{+}^{2}+1}{n_{+}+c}-c)\lambda^{2} & +(\frac{\alpha n_{+}^{3}-c\left(n_{+}^{2}+1\right)}{n_{+}+c}+2w_{+}n_{+}-\delta )\lambda \\ & +\frac{\left(n_{+}^{2}+1\right)\left(2w_{+}n_{+}-\delta \right)-2w_{+}n_{+}^{3}}{n_{+}+c}=0. \end{array}$$ (15)

It is difficulty to obtain the analytical conditions for Hopf bifurcation due to the complexity of the characteristic Eq (15). We provide a bifurcation analysis with biological meaningful parameters by using bifurcation software Matcont6p6.

**Fig 5:**
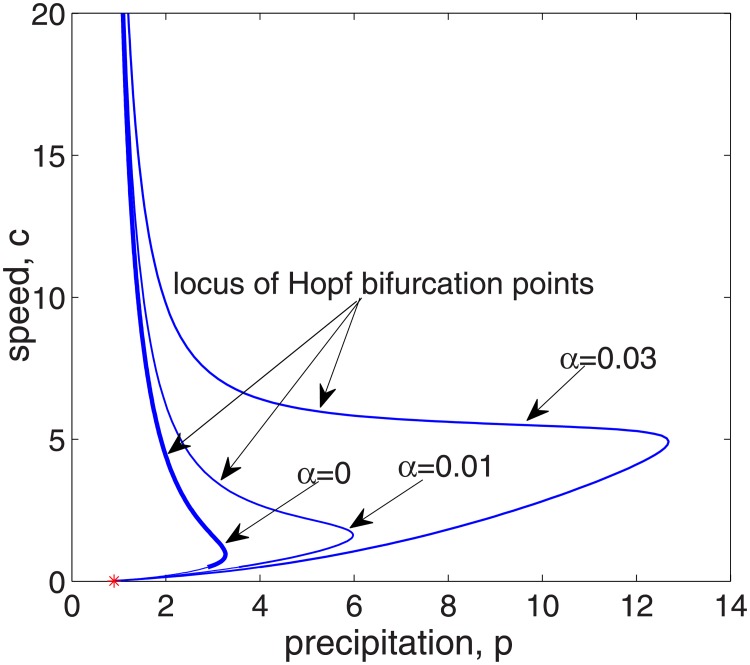
Effects of *α* on the parameter space where periodic pattern solution is predicted by the pattern model (14). The blue lines give the loci of Hopf bifurcation points in *p* − *c* plane with different infiltration feedback levels *α*. The other parameter values are *v* = 182.5, *δ* = 0.45.

Fig 5 presents a bifurcation analysis of system (14) in the *p* − *c* parameter plane. Note that the bifurcation curve with *α* = 0 is the same with the Fig 1 in paper [27] and larger *α* leads to larger region where periodic patterns were emanated. In particular, for fixed value of the migration speed *c*, it can be seen that the range of *p* for pattern formation increases with the increase of infiltration feedback strength *α*, which implies that the infiltration feedback of biomass enlarges the range of *p* in which the vegetation pattern is formed. This confirms the result obtained by linear analysis above in Fig 3. Similarly, for some fixed values of *p*, the range of speed *c* for pattern formation also increases with the increase of infiltration feedback strength of biomass *α*.

**Fig 6:**
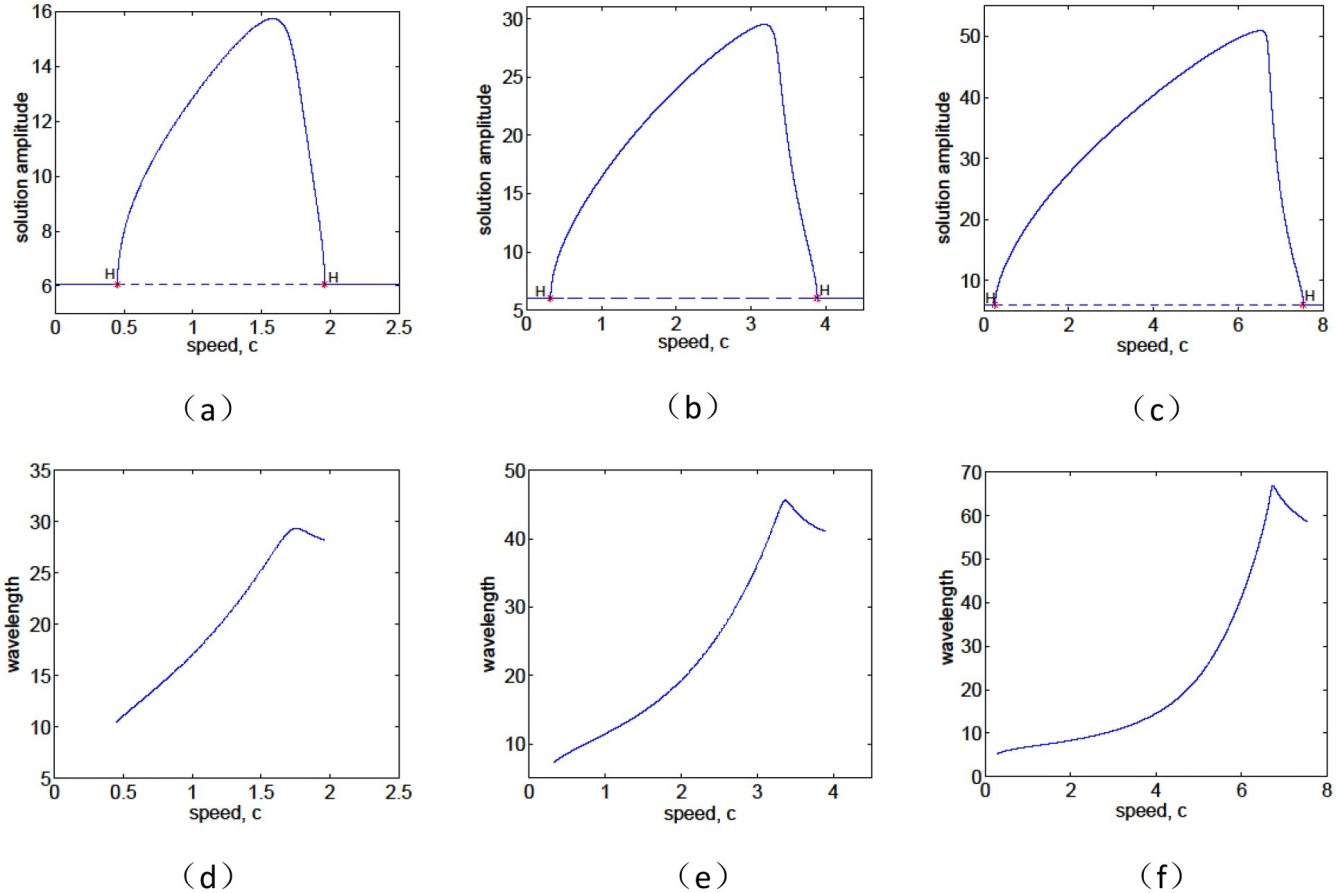
Effects of *α* on the amplitude, wavelength and wave speed of stripe pattern solutions with the parameter values *p* = 2.8, *v* = 182.5 and *δ* = 0.45. (a), (b), (c): typical bifurcation diagrams and spatial solutions of model (14) with (a)*α* = 0, (b)*α* = 0.01 and (c)*α* = 0.03. Here the homogeneous steady state is stable (solid dark line) for large values of the speed *c*, but unstable (dashed line) at intermediate values of *c*. The changes in stability occur via Hopf bifurcations (denoted by H) from which branches of periodic orbits emanate, which indicates that stripe patterns of biomass emerge. (d), (e), (f): the wavelength along the periodic orbit branches varies with different speed.

On the other hand, if the precipitation is fixed at *p* = 2.8, a typical bifurcation diagram for the amplitude and associated wavelength of the spatial solution is given in Fig 6. One can see that there are changes in stability via Hopf bifurcations (denoted by H) at critical value of speed *c*. When the speed *c* is under the critical value, equilibrium remain stable and no periodic solution is formed. With the increase of *c* and the speed *c* is above the critical value, equilibrium stability is lost and a periodic orbit emanates. The periodic orbits are generated by Hopf bifurcation in an intermediate range of speeds and no pattern solutions occur for large speed (Fig 6(a)–6(c)). With the same parameter values as in Fig 6(a)–6(c), we also give the wavelength of the spatial solutions (Fig 6(d)–6(f)), which corresponds to the period of the periodic orbit. It is shown that there is a nonlinear relationship between the wavelength and wave speed. In detail, one can note that the wavelength initially increases with *c* being increased, reaches a maximum, and then decreases. At last, when the wave speed is fixed at the same level (for example *c* = 1), we can see that both the amplitude and the wavelength of pattern solution increase with the increase of infiltration feedback level *α*.

### Patterns with numerical results

In this section, we would like to analyze the numerical solution of system (3) in one-dimensional space. Here we apply finite difference method to the diffusion operator and the convective term and then Euler integration to the finite-difference equations. The simulations reported below are performed on a line spatial grid consisting of 100 cells (100*m*), with the initial conditions consisting of random perturbations about the unstable uniform vegetation state *E*_+_. We assume the simulated domain to be a section of a large biomass field and then it is reasonable to adopted periodic boundary condition.

**Fig 7:**
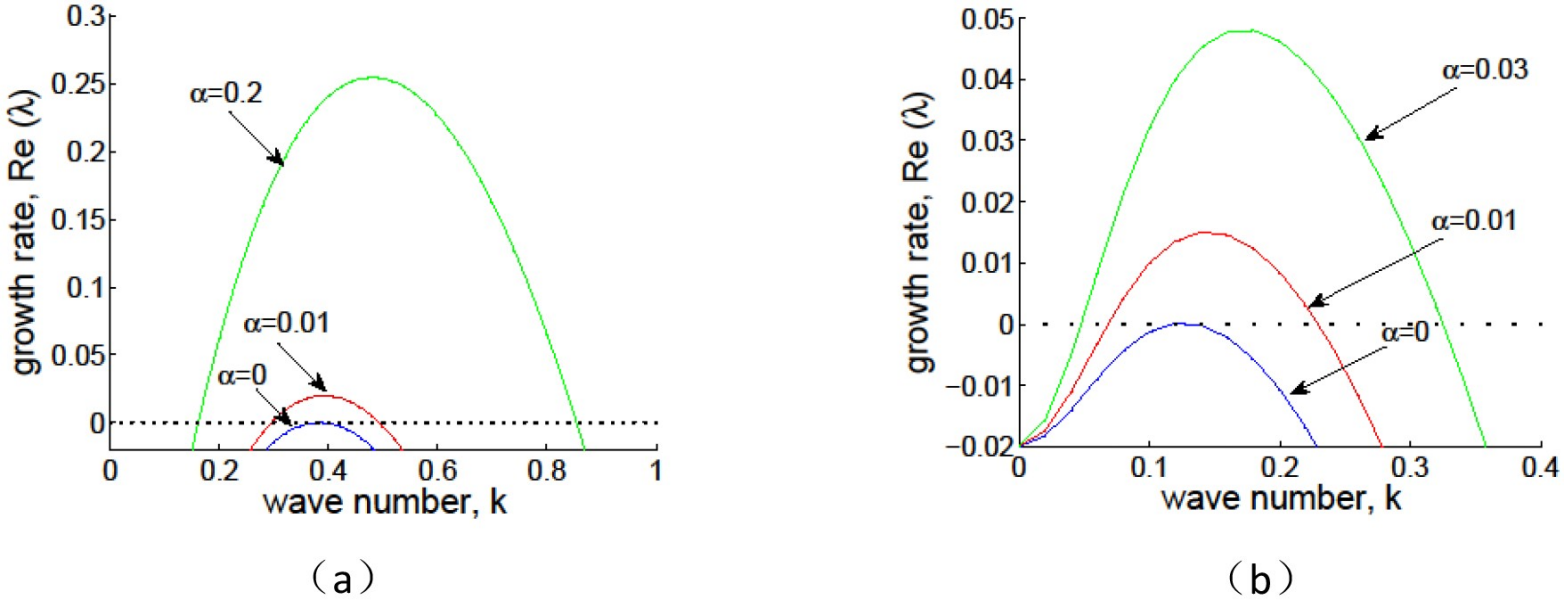
Effect of *α* on the stability of *E*_+_ by the dispersion relation from Eq (9). The parameter values are (a) *p* = 1, *δ* = 0.45, *v* = 9, which corresponds to grass; (b)*p* = 0.1, *δ* = 0.045, *v* = 30, which corresponds to tree.

**Fig 8:**
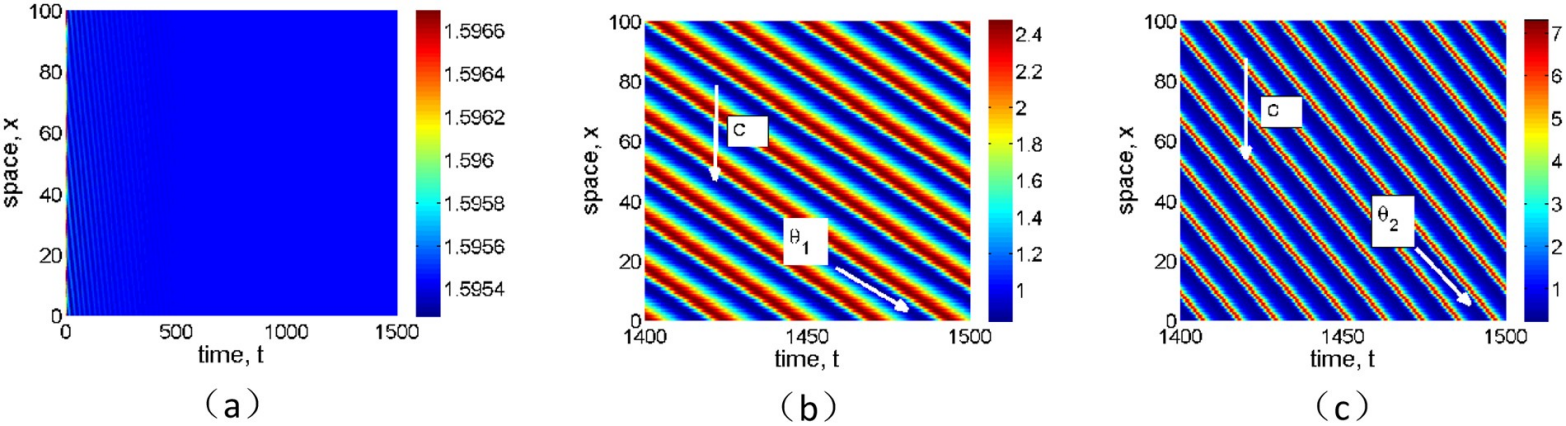
Effects of infiltration positive feedback coefficient *α* on the formation of spatial pattern for grass. The fixed parameter values *p* = 1, *δ* = 0.45, *v* = 9, and (a) *α* = 0, (b) *α* = 0.01 and (c) *α* = 0.2.

**Fig 9:**
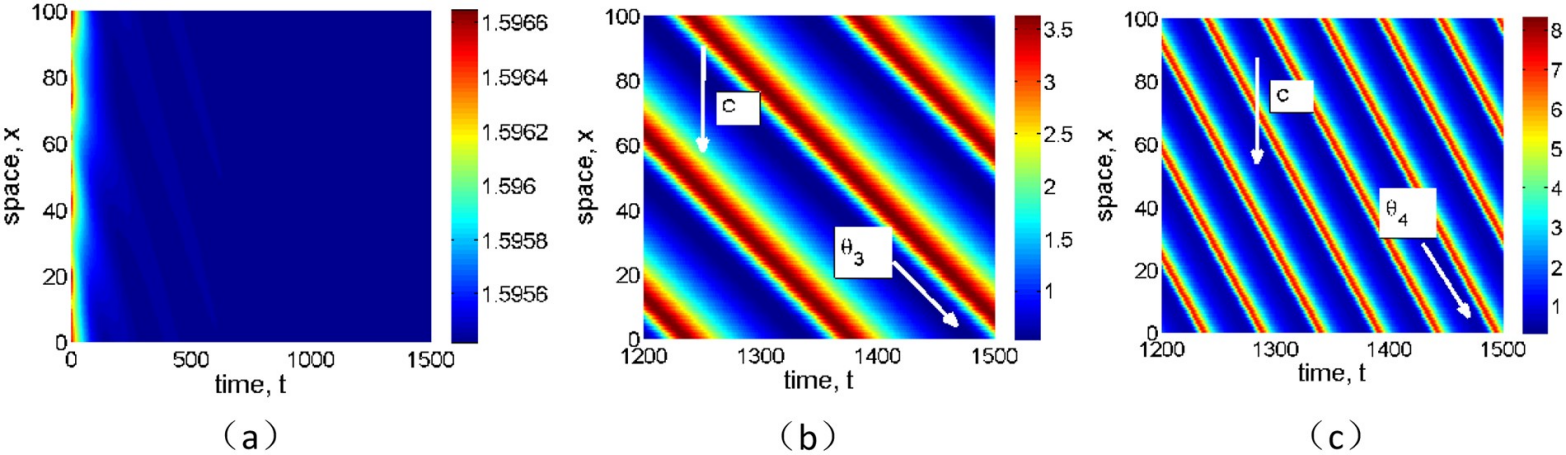
Effects of infiltration positive feedback coefficient *α* on the formation of spatial pattern for tree. The fixed parameter values *p* = 0.1, *δ* = 0.045, *v* = 30, and (a) *α* = 0, (b) *α* = 0.01 and (c) *α* = 0.03.

Fig 7 depicts the effect of *α* on the stability of *E*_+_ obtained by the dispersion relation (9). Following [1], we take *p* = 1, *δ* = 0.45 and *v* = 9 in Fig 7(a), and *p* = 0.1, *δ* = 0.045 and *v* = 30 in Fig 7(b), which corresponds to grass and tree respectively. One can see that there is no vegetation pattern when the positive feedback function is closed (*α* = 0). With the increase of the positive feedback strength (bigger *α*), the maximum value of the growth rate Reλ > 0, which indicates that the patterns formed in Figs 8 and 9 are induced by the infiltration feedback of surface runoff. Then we know that the infiltration feedback can enhance the formation of spatial pattern in this vegetation model, which is consistent with the results above.

The typical traveling spatial patterns for grass and tree are given in Figs 8 and 9 for model (3), which give the effects of infiltration-induced positive feedback *α* on biomass pattern formation. Here the parameter values are the same with those in Fig 7. It can be shown that both the values of the advection speed *v* in Figs 8 and 9 are below critical advection speed as predicted in Fig 2. If the positive feedback function is absent, then no vegetation pattern occur (Figs 8(a) and 9(a)). With the positive feedback function being turned on, traveling spatial patterns occur after some time period (Figs 8(b), 8(c), 9(b) and 9(c)). As we described in the previous section, our numerical results confirm that a stronger positive feedback increases the region where vegetation bands occur.

At the same time, the wave speed of vegetation pattern can be obtained by relationship *c* = |tan*θ*|, where *θ* is the angle of strip as shown in Figs 8(b), 8(c), 9(b) and 9(c). Then we know that the velocity with positive feedback strength *α* = 0.01 equals to *c*_1_ = |tan*θ*_1_|, and the movement velocity with *α* = 0.2 is *c*_2_ = |tan*θ*_2_|. From Fig 8(b) and 8(c), it can be seen that *θ*_2_ > *θ*_1_, which indicates that the movement velocity for grass with *α* = 0.2 is larger than that with *α* = 0.01, that is *c*_2_ > *c*_1_. Similarly, we can observe that *θ*_4_ > *θ*_3_ in Fig 9(b) and 9(c), which indicates that the movement velocity for tree with *α* = 0.03 is larger than that with *α* = 0.01, that is *c*_4_ = |tan*θ*_4_| > *c*_3_ = |tan*θ*_3_|. Then we know that the wave speed of stripe pattern increases with respect to the positive feedback strength *α*.

**Fig 10:**
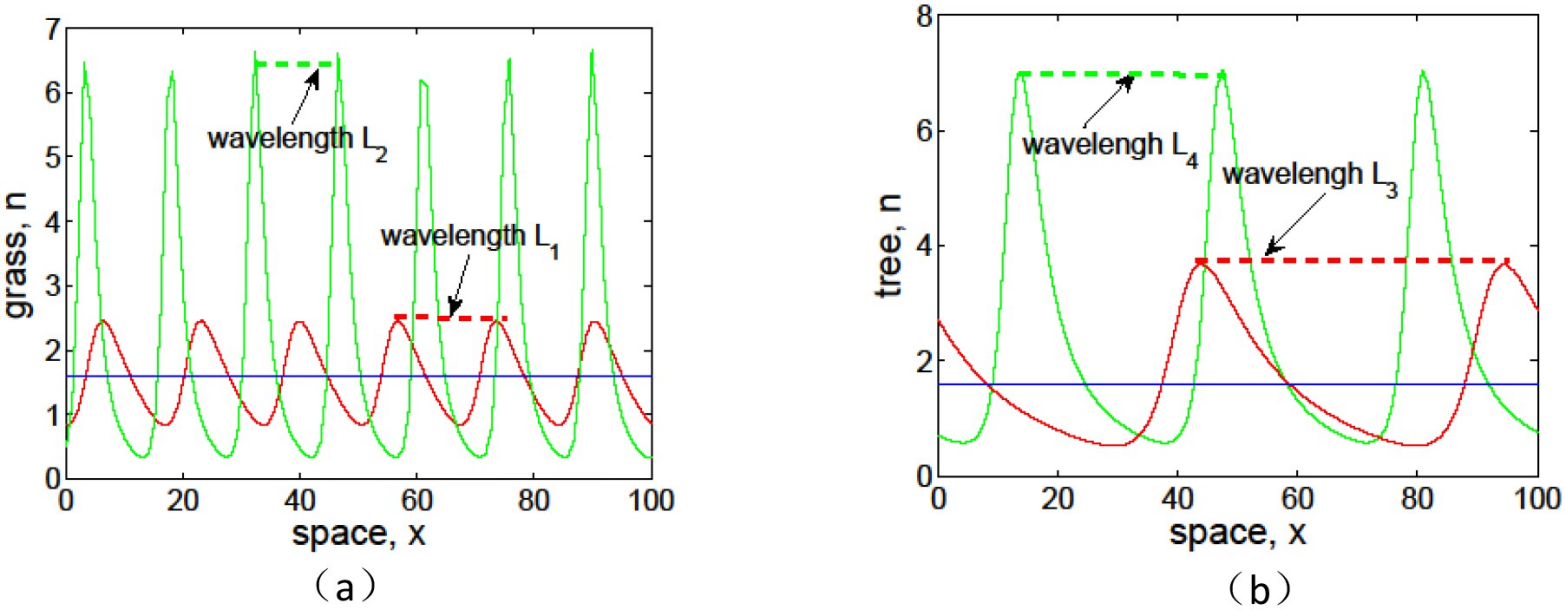
Effects of infiltration positive feedback coefficient *α* on the amplitude and wavelength of pattern solutions for biomass *n*. The blue curves correspond to *α* = 0, red curves correspond to *α* = 0.01 in (a) and (b), and the green curves correspond to *α* = 0.2 in (a) and *α* = 0.03 in (b). The fixed parameter values: (a) *p* = 1, *δ* = 0.45, *v* = 9, which corresponds to grass. (b) *p* = 0.1, *δ* = 0.045, *v* = 30, which corresponds to tree.

Fig 10 is a cross-sectional drawing of Figs 8(b), 8(c), 9(b) and 9(c) at *t* = 1500, which describes the effects of infiltration-induced positive feedback *α* on the amplitude and wavelength of pattern solutions for biomass *n*. It can be seen that the stronger positive feedback leads to larger amplitude for both grass and tree. At the same time, noting that *L*_1_ > *L*_2_ in Fig 10(a) and *L*_3_ > *L*_4_ in Fig 10(b), we know that both the wavelengths of banded vegetation patterns for grass and tree are negatively correlated with *α*, which is consistent with the results as predicted in linear analysis in Fig 2. Especially, comparing Fig 10(a) with Fig 10(b), we see that *L*_3_ > *L*_4_ > *L*_1_ > *L*_2_ and *L*_3_ − *L*_4_ > *L*_1_ − *L*_2_, which indicate that the wavelength of trees is larger than that of grass and the trees are more sensitive to the variation of *α* than grasses.

**Fig 11:**
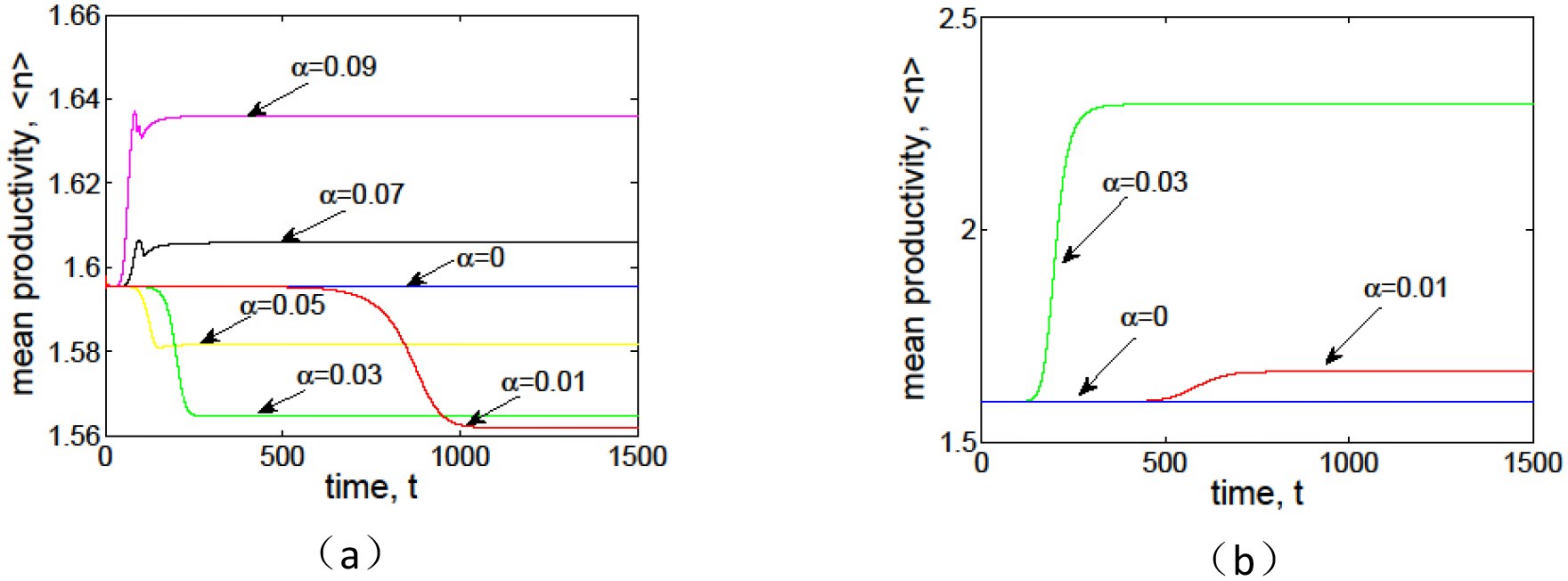
Effects of infiltration positive feedback coefficient *α* on mean productivity of vegetation pattern in arid area. The fixed parameter values (a) *p* = 1, *δ* = 0.45, *v* = 9, which corresponds to grass; (b) *p* = 0.1, *δ* = 0.045, *v* = 30, which corresponds to tree.

It is also interesting to compare the mean productivity with different positive feedback levels. Fig 11 gives an illustration of the mean productivity of grass and tree with different positive feedback levels *α*, where the blue lines represent the values of homogeneous state. We can see that the mean productivity of vegetation pattern increases with stronger infiltration-induced positive feedback. In particular, the mean productivity of grass pattern is lower than that of homogeneous steady state when positive feedback is relatively weak and it is larger than that with stronger positive feedback (Fig 11(a)). On the other hand, the mean productivity of tree pattern with positive feedback *α* = 0.03 is always larger than that with *α* = 0.01, as well as the mean production for homogeneous steady state (Fig 11(b)).

## Discussion

In this paper, we investigated the effects of infiltration feedback of surface runoff on the formation and characteristics of banded vegetation pattern on hillsides of semiarid area. In fact, the formation and characteristics of spatial stripe pattern may be affected by some other factors in ecological systems, which include vegetation-climate feedback, circumstance noise and the micro-scale vegetation moisture feedback. It is also important and interesting to investigate the effects of these factors on the formation and characteristics of banded vegetation pattern.

## Conclusion

This paper investigated the influence of infiltration feedback on the characteristic of banded vegetation pattern on hillsides of semiarid area based on an extensive model of the classical Klausmeier′s model [1]. We have presented a detailed linear stability analysis and validated our theoretical findings through detailed parameter space identification and numerically solving the model. Although the current model greatly simplifies the biophysics of arid systems, some interesting results can be derived from this simple model.

Firstly, it is shown that there is no Turing instability range in system (3) and the introduction of the infiltration feedback of the surface runoff leads to smaller critical slope and larger instability region in *p* − *δ* plane, witch implies that the infiltration feedback improve the formation of vegetation pattern(see Figs 2 and 3). It can be found that the patterns formed within the reasonable parameter space are periodic both in space and time (see Fig 4). It is also shown that the wavelength of stripe pattern decreases as infiltration feedback coefficient *α* is increased (see Fig 10). On the other hand, both the wave speed and amplitude of solutions increase with the introduction of the positive feedback function of infiltration(see Figs 8, 9 and 10). At last, we find that the mean production of grass is lower than that of homogeneous steady state when infiltration feedback is relatively weak and it is larger than that with stronger positive feedback (see Fig 11(a)). On the other hand, the mean productivity of tree pattern with stronger positive feedback is always larger than that with weaker positive feedback, as well as the mean production for homogeneous steady state (Fig 11(b)).

## Supporting information

S1 Appendix Proof of proposition 0.2. (PDF)
