# Supplementary material for: The influence of infiltration feedback on the characteristic of banded vegetation pattern on hillsides of semiarid area
Source: PLoS One. 2019 Jan 15;14(1):e0205715. doi: 10.1371/journal.pone.0205715 (PMC6333328; doi:10.1371/journal.pone.0205715)
Supplement: S1 Appendix — (PDF) [file pone.0205715.s001.pdf]

The influence of infiltration feedback on the characteristic of banded vegetation pattern on hillsides of semiarid area

Xiaoli Wang[1,◑], Guohong Zhang[1*,◑],

1 School of Mathematics and Statistics, Southwest University, Chongqing, 400700, P.R.China

◑These authors contributed equally to this work.
* corresponding author: Guohong Zhang

S1 Appendix. Proof of Theorem 0.2

proof: It follows that E_+ is locally asymptotically stable if and only if

$$\delta - 1 - n_+^2 < 0. \tag{1}$$

If $\delta < 1$, it is easy to see that the inequality (1) holds, which indicates that E_+ is locally asymptotically stable.

If $\delta > 1$, it can be seen that (1) holds if and only if $n_+ > \sqrt{\delta - 1}$, which is equivalent to

$$\sqrt{p^2 - 4\delta^2} > 2\delta\sqrt{\delta - 1} - p. \tag{2}$$

Condition (2) apparently holds if

$$p > 2\delta\sqrt{\delta - 1}. \tag{3}$$

It can be shown that $2\delta\sqrt{\delta-1} > 2\delta$ if and only if $\delta > 2$. When $\delta < 2$, we have $2\delta\sqrt{\delta-1} < 2\delta$, which implies that E_+ is locally asymptotically stable.

When $\delta > 2$, we have $2\delta\sqrt{\delta-1} > 2\delta$. If $2\delta < p < 2\delta\sqrt{\delta-1}$, it follows that (2) holds if

$$p > \frac{\delta^2}{\sqrt{\delta - 1}}. \tag{4}$$

It follows from (3) and (4) that (1) holds if $p > \overline{p}$, which means that E_+ is locally asymptotically stable.

On the other hand, if $\delta > 2$ and $p < \overline{p}$, we have $\delta - 1 - n_+^2 > 0$, which indicates that E_+ is unstable.

Set $T(p) = \mathrm{tr}(J_{E_+})$ and $D(p) = \mathrm{deb}(J_{E_+})$. We can prove that $p = \widehat{p}$ is a Hopf bifurcation point if $\delta > 2$. We should identify

$$T(\overline{p}) = 0, \quad D(\overline{p}) \neq 0, \quad \alpha'(\overline{p}) \neq 0, \tag{5}$$

where $\alpha(p) \pm i\beta(p)$ is the unique pair of complex eigenvalues near the imaginary axis.

It is easy to verify that $T(\overline{p}) = 0$ and $D(\overline{p}) \neq 0$ from the proof above. Noting that $\alpha(p) \pm i\beta(p)$ are the eigenvalues of $J(E_+)$, we have

$$\alpha(p) = \frac{T(p)}{2}, \quad \beta(p) = \frac{\sqrt{4D^2(p) - T^2(p)}}{2}.$$

Then we obtain

$$\alpha'(\overline{p}) = -\frac{(\overline{p} + \sqrt{\overline{p}^2 - 4\delta^2})n_+}{\delta\sqrt{\overline{p}^2 - 4\delta^2}} < 0.$$

Hence the transversality condition in (5) is also satisfied. It follows from the well-known Hopf bifurcation results that $p = \overline{p}$ is a Hopf bifurcation point where a family of small amplitude limit cycles emanate from E_+. The proof is completed.
